# Explaining Maternal Preventive Behaviors for Home Accident Risk in Children Under Five Years: An Application of the Health Belief Model

**DOI:** 10.34172/hpp.45257

**Published:** 2026-06-06

**Authors:** Nasrin Beyzaei Nasrabadi, Mohammad Ali Morowatisharifabad

**Affiliations:** ^1^Department of Health Education and Promotion, School of Public Health, Shahid Sadoughi University of Medical Sciences, Yazd, Iran; ^2^Elderly Health Research Center, School of Public Health, Shahid Sadoughi University of Medical Sciences, Yazd, Iran; ^3^Department of Aging and Health, School of Public Health, Shahid Sadoughi University of Medical Sciences, Yazd, Iran

**Keywords:** Accidents, Home, Child, Health Belief model, Locus of control, Mothers, Preventive behavior

## Abstract

**Introduction:**

Home accidents are a major global health concern for children under five. This study aimed to determine the prevalence of home accidents and to identify the predictors of mothers’ preventive behaviors based on the Health Belief Model (HBM) in Yazd, Iran.

**Methods:**

This cross-sectional study was conducted in 2023 on 200 mothers with children under five years, selected via multi-stage cluster sampling from five comprehensive health centers in Yazd. A researcher-made questionnaire based on HBM, including locus of control, was used for data collection. Its validity and reliability were confirmed (CVR>0.75, CVI>0.79, Cronbach’s alpha>0.68). Data were analyzed using SPSS-21 with independent t-tests, ANOVA, Pearson correlation, and linear regression, considering the design effect.

**Results:**

Totally, of 212 studied children, 163 (76.9%, 95% CI: 71.2-82.6) had experienced at least one home accident. The most common accidents were cuts (39.6%), falls (33.0%), and burns (30.7%). Accident occurrence was significantly associated with older child age (mean difference: 8.25 months, 95% CI: 3.38-13.12, *P*=0.005) and higher household income (*P*=0.008). Mothers of children without accidents had significantly higher self-efficacy (mean difference: 5.49, 95% CI: 1.48-9.50, *P*=0.018), lower perceived barriers (mean difference: -5.92, 95% CI: -10.84 to -1.00, *P*=0.027), and higher preventive behavior scores (mean difference: 1.78, 95% CI: 0.72-2.84, *P*=0.017). Linear regression showed that perceived barriers (β=-0.297, *P*<0.001), perceived benefits (β=0.250, *P*<0.001), powerful others locus of control (β=-0.226, *P*<0.001), and chance locus of control (β=-0.156, *P*=0.019) were significant predictors, explaining 35.8% of the variance in preventive behaviors.

**Conclusion:**

The HBM was found to be an effective framework for explaining maternal preventive behaviors. Interventions should focus on reducing perceived barriers, enhancing perceived benefits, and modifying external locus of control beliefs to prevent home accidents in children.

## Introduction

 Childhood injuries are associated with significant rates of disability and mortality worldwide.^1^ According to the World Health Organization (2022), approximately 830,000 children under 18 years die annually from unintentional injuries, with over 200,000 cases occurring in children under five years old.^1^ Although children spend more than half of their time at home before reaching school age, they face higher injury risks compared to school-aged children, making the home environment a major contributor to childhood accidents.^2^

 Unintentional home accidents are defined as injuries occurring within or around the home environment.^3^ The Iranian Legal Medicine Organization (2023) reports that more than 3,000 children under five years die annually from unintentional injuries in Iran, with falls, drowning, and burns being the most common causes of mortality in this age group.^4^ These statistics highlight the urgent need for effective prevention strategies tailored to the Iranian context.

 Preventing childhood injuries across all age groups is crucial to reducing mortality among children who have survived other early-life diseases.^5^ Current preventive measures against childhood injuries remain fragmented and insufficient, despite accompanying educational programs.^6^ Comprehensive planning requires detailed information about injury patterns, and maternal education plays a pivotal role in family-based prevention.^7^ Therefore, baseline assessments of maternal knowledge and attitudes are necessary, highlighting the need for theory-based behavioral studies.^8^

 The Health Belief Model (HBM) provides a valuable theoretical framework for injury prevention strategies.^9^ As a predictive theory of health behavior, HBM’s perceived susceptibility and severity constructs help individuals recognize health threats, while perceived benefits, perceived barriers, self-efficacy, and cues to action influence preventive behaviors.^10^ The model also incorporates health locus of control—the belief that health maintenance depends either on personal actions (internal control) or external factors (external control).^11^ Parents with stronger internal locus of control are more likely to implement effective injury prevention measures for their children,^12^ and HBM-based health education can enhance participants’ knowledge, modify health beliefs, and improve preventive behaviors.^13^

 Existing Iranian studies on home accidents have primarily focused either on prevalence surveys or examined limited factors without theoretical foundations.^14^ Few investigations have used behavioral theories to predict home accident patterns. To the best of our knowledge, based on a systematic search of databases including PubMed, Scopus, and the Scientific Information Database (SID) up to 2023, no previous study in Iran has assessed the determinants of home accident prevention behaviors among mothers using the full HBM, including the locus of control construct. Given the magnitude of home accidents in Iran and the importance of prevention, this study examines home accidents in children under five years and identifies their mothers’ preventive behavior determinants using the comprehensive HBM framework.

## Methods

###  Study Design and Participants

 This cross-sectional study was conducted in 2023 in urban comprehensive health care centers (CHCCs) in Yazd, Iran. CHCCs are first-level health care providers within the Iranian primary health care system, offering preventive care and maternal-child health services. To account for the hierarchical structure of the population, a multistage cluster sampling method was employed. Initially, five centers were randomly selected from all 19 urban CHCCs in Yazd using a simple random sampling technique. Subsequently, a list of all registered children under five years was extracted from each selected center via the SIB (integrated electronic health system in Iran). From these lists, 40 eligible mothers per center were selected using a computer-based random number generator, resulting in a total sample of 200 mothers. The sample size was determined based on a similar study, considering a 99% confidence level and a design effect of 1.1 to ensure adequate precision and compensate for potential non-response. Equal allocation of participants across clusters minimized variance inflation due to unequal cluster sizes. Inclusion criteria were: Iranian nationality, having at least one child under five years of age (up to 4 years, 11 months, and 29 days), having an active electronic health record in the SIB system, minimum literacy to comprehend and complete the questionnaire, permanent residence in Yazd during the study period, and providing written informed consent. Mothers who declined to participate or returned incomplete questionnaires were excluded.

###  Instruments

 A researcher-developed questionnaire based on HBM was used, consisting of four main sections. The first section focused on demographics and background (17 items). The second section assessed the history of home accidents. The third section included HBM constructs: Knowledge (9 items, 3-option), Perceived Susceptibility (17 items, α = 0.857), Perceived Severity (11 items, α = 0.953), Perceived Benefits (3 items, α = 0.819), Perceived Barriers (11 items, α = 0.792), Cues to Action (7 items, α = 0.797), Self-efficacy (3 items, α = 0.940), and Locus of Control (9 items covering internal, powerful others, and chance domains, α = 0.681). All constructs except knowledge were measured on a 5-point Likert scale. The final section focused on preventive behaviors, comprising 31 questions (17 yes/no and 14 Likert-scale items). The psychometric evaluation of the questionnaire was conducted by a panel of 8 health education and promotion experts, yielding a Content Validity Ratio (CVR) and Content Validity Index (CVI) above 0.75 and 0.79, respectively. Based on expert feedback, two knowledge questions and one perceived susceptibility question were removed. Reliability was further confirmed in a pilot study with 15 mothers, where the alpha coefficient for behaviors was 0.657, and test-retest reliability (ICC) for knowledge and behaviors was 0.80 and 0.95, respectively.

###  Outcome Measures

 The primary outcome was the maternal preventive behavior score regarding home accident prevention. Secondary outcomes were the occurrence of home accidents in children under five and the scores of various HBM constructs.

###  Statistical Analysis

 Data analysis was performed using SPSS version 21 (IBM Corp., Armonk, NY, USA). To account for the multi-stage sampling design, the Complex Samples module was used, applying sampling weights. Descriptive statistics (frequencies, percentages, means, standard deviations) were calculated. Normality of continuous data was assessed using the Kolmogorov-Smirnov test (*P* > 0.05). Independent t-tests were used to compare HBM construct scores between the accident and no-accident groups. One-way ANOVA was used for comparisons involving more than two groups. Pearson correlation coefficients were calculated for bivariate relationships. A multivariable linear regression analysis (enter method) was performed to identify predictors of preventive behaviors. Assumptions of linear regression (linearity, normality of residuals, homoscedasticity, and multicollinearity) were checked and met. A P-value of less than 0.05 was considered statistically significant. Missing data were minimal ( < 2%) and handled by listwise deletion.

## Results

 A total of 200 mothers with 212 children under five years participated. The mean age of mothers was 36.0 ± 5.0 years. Most mothers were homemakers (67.0%) and held a bachelor’s degree (37.0%). The mean age of the children was 36.0 ± 5.1 months; 53.8% were boys, and 43.9% were second-born children. Full demographic details are presented in Table 1.

**Table 1:** Demographic characteristics of study participants (n = 200 mothers, n = 212 children)

**Variable**	**Category**	**n**	**% (95% CI)**
Child sex	Female	98	46.2 (39.5-53.0)
	Male	114	53.8 (47.0-60.5)
Child age (months)	< 12	23	10.8 (6.7-15.0)
	13-24	35	16.5 (11.5-21.5)
	25-36	56	26.4 (20.5-32.4)
	37-48	61	28.8 (22.7-34.9)
	49-60	37	17.5 (12.4-22.6)
Birth order	First child	38	17.9 (12.7-23.1)
	Second child	93	43.9 (37.2-50.6)
	Third child or higher	81	38.2 (31.6-44.8)
Maternal education	Middle school or lower	20	10.0 (5.8-14.2)
	High school diploma	58	29.0 (22.7-35.3)
	Associate degree	20	10.0 (5.8-14.2)
	Bachelor's degree	74	37.0 (30.3-43.7)
	Master's degree or higher	25	12.5 (7.9-17.1)
	PhD or higher	3	1.5 (0-3.2)
Paternal education	Middle school or lower	32	16.0 (10.9-21.1)
	High school diploma	68	34.0 (27.4-40.6)
	Associate degree	20	10.0 (5.8-14.2)
	Bachelor's degree	34	17.0 (11.8-22.2)
	Master's degree or higher	9	4.5 (1.6-7.4)
	PhD or higher	37	18.5 (13.1-23.9)
Maternal employment	Homemaker	134	67.0 (60.5-73.5)
	Employed	46	23.0 (17.2-28.8)
	Student	8	4.0 (1.3-6.7)
	Other	12	6.0 (2.7-9.3)
Family income level	Low	15	7.5 (3.8-11.2)
	Medium	105	52.5 (45.6-59.4)
	High	68	34.0 (27.4-40.6)
	Very high	12	6.0 (2.7-9.3)
Housing status	Rental	60	30.0 (23.6-36.4)
	Private	140	70.0 (63.6-76.4)
Childcare arrangement	Daycare	23	11.5 (7.1-15.9)
	With nanny at home	13	6.5 (3.1-9.9)
	With father at home	13	6.5 (3.1-9.9)
	With relatives	36	18.0 (12.7-23.3)
	With mother	112	56.0 (49.1-62.9)
	Other	3	1.5 (0-3.2)

 Of the 212 children, 163 (76.9%, 95% CI: 71.2-82.6) had experienced at least one home accident. Home accidents such as burns (58.1%), cuts (39.6%) and falls (33%) were most common in children under five years. The kitchen (36.54%), living room (27.13%) and bedroom (17.48%) were the most common locations for home accidents in children under five years. Most accidents resulted in recovery (98.17%).

 Accident occurrence was significantly associated with the child’s age. Children who experienced an accident were older (mean age 38.1 ± 13.7 months) than those who did not (29.9 ± 18.2 months, mean difference = 8.3 months, 95% CI: 3.4-13.1, *P* = 0.005). The highest frequency of accidents was in the 37-48 month age group (33.1%).

 A significant association was also found with household income (*P* = 0.008). Interestingly, the proportion of children with accidents increased with income level, from 58.8% in the low-income group to 91.7% in the very high-income group (Table 2).

**Table 2:** Frequency of home accidents by household income level

**Income level**	**Total Children (n)**	**Accidents n (%)**	**No Accidents n (%)**
Low	17	10 (58.8)	7 (41.2)
Medium	105	75 (71.4)	30 (28.6)
High	68	61 (89.7)	7 (10.3)
Very high	12	11 (91.7)	1 (8.3)
**Total**	**212**	**163 (76.9)**	**49 (23.1)**

*Pearson chi-square (3) = 11.8, *P* = 0.008*

 Among home environment features, the presence of a balcony was significantly associated with a higher rate of accidents (*P* = 0.002), particularly falls and fractures. No significant associations were found with child’s gender, mother’s employment status, or other home environment conditions.

 The ranges and mean scores for all HBM constructs are presented in Table 3.

**Table 3:** Range and mean scores of HBM constructs (n = 200 mothers)

**Construct**	**Min**	**Max**	**Mean±SD**	**95% CI**
Knowledge	0	9	4.91 ± 1.99	4.63-5.19
Perceived Susceptibility	14	60	46.59 ± 8.00	45.48-47.70
Perceived Severity	11	68	34.86 ± 9.41	33.55-36.17
Perceived Benefits	11	25	21.84 ± 3.11	21.41-22.27
Perceived Barriers	11	55	28.52 ± 8.08	27.40-29.64
Cues to Action	5	20	15.89 ± 2.83	15.50-16.28
Self-efficacy	9	25	18.31 ± 3.91	17.77-18.85
Internal Locus of Control	6	15	11.87 ± 2.11	11.58-12.16
Powerful Others Locus	3	15	10.95 ± 2.14	10.65-11.25
Chance Locus of Control	3	15	8.76 ± 2.61	8.40-9.12
Preventive Behaviors	37	82	65.12 ± 9.95	63.74-66.50

 Significant differences were observed between mothers of children with and without accidents for three constructs (Table 4). Mothers of children without accidents had significantly higher self-efficacy, lower perceived barriers, and higher preventive behavior scores.

**Table 4:** Comparison of HBM constructs between mothers of children with and without accidents

**Construct**	Accident Group (n=163 mothers*) Mean±SD	No Accident Group (n=49 mothers*) Mean±SD	**Mean Difference (95% CI)**	***P*** ** value**
Self-efficacy	14.00 ± 5.00	19.49 ± 3.41	5.49 (1.48, 9.50)	0.018
Perceived Barriers	28.20 ± 8.11	22.28 ± 4.03	-5.92 (-10.84, -1.00)	0.027
Preventive Behaviors	64.22 ± 9.84	68.10 ± 9.75	1.78 (0.72, 2.84)	0.017
Internal Locus of Control	11.80 ± 2.18	12.10 ± 1.82	-0.30 (-0.97, 0.37)	0.584
Powerful Others Locus	10.90 ± 2.24	11.10 ± 1.85	-0.20 (-0.88, 0.48)	0.380
Chance Locus of Control	8.47 ± 2.40	8.85 ± 2.20	-0.38 (-1.12, 0.36)	0.823
Perceived Severity	54.38 ± 9.30	55.40 ± 8.81	-1.02 (-3.92, 1.88)	0.750
Perceived Benefits	21.73 ± 3.17	22.24 ± 2.91	-0.51 (-1.49, 0.47)	0.311
Cues to Action	15.76 ± 2.91	16.34 ± 2.52	-0.58 (-1.47, 0.31)	0.205
Knowledge	4.86 ± 1.91	5.08 ± 2.24	-0.22 (-0.90, 0.46)	0.506

*Note*: n represents number of mothers, as some mothers had more than one child. Analysis was performed at the mother level for HBM constructs.

 Maternal education level was significantly associated with both knowledge (*P* = 0.006) and perceived susceptibility (*P* = 0.014) (Table 5).

**Table 5:** Mean ± SD scores of knowledge and perceived susceptibility by maternal education level

**Maternal education**	**n**	**Knowledge (Mean±SD)**	**Perceived Susceptibility (Mean±SD)**
Middle school or lower	20	3.45 ± 2.21	44.90 ± 7.34
High school diploma	58	4.73 ± 2.02	44.78 ± 8.15
Associate degree	20	5.04 ± 1.68	49.76 ± 7.46
Bachelor's degree	74	5.12 ± 1.92	46.25 ± 7.60
Master's degree or higher	25	5.66 ± 1.79	50.51 ± 8.46
PhD or higher	3	5.00 ± 1.00	46.33 ± 4.72
*P* value		0.006	0.014

###  Predictors of Preventive Behaviors

 The multivariable linear regression model showed that HBM constructs significantly predicted mothers’ preventive behaviors (*P* < 0.001), explaining 35.8% of the variance (adjusted R^2^ = 0.342). The results are presented in Table 6.

**Table 6:** Multiple linear regression analysis for predictors of preventive behaviors

**Predictor**	**Standardized Beta (β)**	***P*** ** value**
Knowledge	0.097	0.139
Perceived Susceptibility	-0.055	0.396
Perceived Severity	0.068	0.280
Perceived Benefits	0.250	< 0.001
Perceived Barriers	-0.297	< 0.001
Cues to Action	0.122	0.077
Self-efficacy	0.092	0.162
Internal Locus of Control	0.094	0.208
Powerful Others Locus	-0.226	< 0.001
Chance Locus of Control	-0.156	0.019

*R^2^ = 0.358, Adjusted R^2^ = 0.342*

 Perceived barriers (β = -0.297) was the strongest negative predictor, followed by powerful others locus of control (β = -0.226) and chance locus of control (β = -0.156). Perceived benefits (β = 0.250) was a significant positive predictor. Knowledge, perceived susceptibility, severity, cues to action, self-efficacy, and internal locus of control were not significant in the final model.

## Discussion

 This study aimed to assess the prevalence of home accidents among children under five and identify determinants of preventive behaviors in their mothers using the HBM. Findings revealed that most children experienced at least one home accident, highlighting an urgent need for household- and community-level interventions.

 Burns, cuts, and falls were the most frequent home accidents. Geographic variations were evident when comparing studies: Poorolajal et al.^15^ reported falls, burns, and poisoning as predominant; Vakili et al.^16^ noted burns, falls, and poisoning; Rahimi et al.^14^ identified hot liquid burns, thermal device burns, falls, and drug poisoning. Greek data^17^ showed falls, severe injuries, and burns, while the Turkish research^18^ reported falls, poisoning, and burns. A U.S. study^13^ documented falls as the leading cause (76%). These disparities underscore the need for region-specific prevention strategies, though burns and falls remain universally prevalent.

 Most accidents occurred in kitchens, living rooms, and bedrooms, aligning with Mahdizadeh-Esfanjani et al.’s findings^2^ (living rooms, kitchens, courtyards). Balconies emerged as a significant risk factor, emphasizing the need for safety measures (e.g., window guards, cabinet locks).

 Children aged 37–48 months (3–4 years) were most affected, likely due to increased mobility and curiosity, consistent with Poorolajal et al.^15^

 No association was found between accident occurrence and child gender, parental employment, or home environment (except balconies), suggesting cultural/behavioral factors (e.g., inadequate supervision) may outweigh physical household features. However, conflicting results exist: Tsoumakas et al.^17^ linked male gender and maternal employment to higher accident rates, while Aktürk and Erci^18^ found higher rates among children of stay-at-home mothers. Home size ( < 2 bedrooms) and grandparental care reduced accidents in Tsoumakas et al.’s study.^17^

 Paradoxically, higher household income correlated with more accidents in Yazd, contrary to findings from Turkey.^18^ This might be explained by greater access to hazardous items (e.g., electronics, non-standard toys) in higher-income households in this specific cultural setting, or possibly different supervision patterns.^18^ Future qualitative research should explore this unexpected relationship.

 Key HBM constructs demonstrated significant predictive power. Mothers with higher self-efficacy in home safety had significantly fewer accidents. Self-efficacy—belief in one’s ability to implement preventive measures (e.g., installing locks)—was pivotal, aligning with Dehghani et al.^19^ In contrast, Schilling et al.^12^ emphasized locus of control, suggesting cultural influences on psychological constructs.

 Higher maternal education correlated with greater risk awareness and perceived severity of accidents, prompting stricter preventive actions (e.g., keeping hot tea away). However, perceived barriers (cost/time for safety modifications) often hindered implementation, highlighting the gap between knowledge and action. Interactive education (e.g., videos of real-life consequences) could bridge this gap by simultaneously boosting awareness, severity perception, and self-efficacy. This finding is consistent with Lafta et al.^20^ who demonstrated a positive association between maternal knowledge and reduced home accidents.

 Regression analysis revealed that external locus of control (both ‘powerful others’ and ‘chance’ dimensions), perceived barriers, and perceived benefits were key behavioral predictors, explaining 35.8% of the variance. The strong negative influence of perceived barriers and external locus of control suggests that mothers who feel prevention is outside their control or too difficult to implement are less likely to adopt safety behaviors. This aligns with Dehghani et al.^19^ who also identified barriers and self-efficacy as predictors, confirming the HBM’s utility in accident prevention. The prominence of external locus of control in this sample may reflect cultural attitudes towards fate or the influence of family members (‘powerful others’) on maternal safety practices.

## Limitations

 This study has several limitations. First, its cross-sectional design precludes causal inferences. Second, data were based on self-report, which is subject to recall and social desirability bias. Third, the study was conducted in a single city, limiting generalizability. Fourth, while a multi-stage cluster sampling method was employed, standard linear regression was used for analysis. Future studies with more diverse populations are recommended.

## Conclusion

 This study confirms a high prevalence of home accidents among children under five in Yazd and validates the HBM, particularly the constructs of perceived barriers, benefits, and external locus of control, as a useful framework for understanding maternal preventive behaviors. Interventions to reduce home accidents should adopt a multi-pronged approach: (1) implement targeted educational programs to enhance perceived benefits and address fatalistic beliefs associated with external locus of control; (2) develop practical strategies to reduce perceived barriers, such as subsidizing safety devices or providing easy-to-implement safety checklists; and (3) enforce environmental modifications, particularly for high-risk areas like balconies. Cross-sectoral collaboration between the health system, municipal authorities, and community organizations is essential for creating safer home environments for young children.

## Competing Interests

 The authors declare that they have no competing interests.

## Ethical Approval

 The study protocol was approved by the Ethics Committee of Shahid Sadoughi University of Medical Sciences, Yazd, Iran (Ethics Code: IR.SSU.SPH.REC.1403.04). Written informed consent was obtained from all participants after a full explanation of the study’s objectives and assurance of data confidentiality.

## References

[1] World Health Organization. Child Injuries and Violence [Internet]. Geneva: WHO; 2022. Available from: https://www.who.int/news-room/fact-sheets/detail/child-injuries-and-violence. Accessed July 10, 2024.

[2] Mehdizadeh Esfanjani R, Sadeghi-Bazargani H, Golestani M, Mohammadi R (2017). Domestic injuries among children under 7 years of age in Iran; the baseline results from the Iranian First Registry. Bull Emerg Trauma.

[3] Khan S, Tauheed N, Nawab S, Afzal S, Khalique N (2019). Domestic accidents among under-5 year children: a study on the modern day epidemic. Int J Community Med Public Health.

[4] Legal Medicine Organization of Iran. Annual Report on Unintentional Child Mortality Statistics [Internet]. Tehran: LMO; 2023. Available from: http://www.lmo.ir. Accessed July 10, 2024.

[5] World Health Organization. Profile of Child Injuries: Selected Member States in the Asia-Pacific Region. New Delhi: WHO Regional Office for South-East Asia; 2010.

[6] Khodadadi H, Asadpour M, Kermani Z, Ravari A (2006). Frequency of the accidents in children under 15 years old referring to the emergency center of Ali Ebn Abitaleb hospital in Rafsanjan 2000-2001. J Rafsanjan Univ Med Sci.

[7] Rostami H, Mirzaie M, Lotfi SA, Moayyed H, Mazaheri A, Mirzaie A (2006). Home eye trauma in emergency units. Iran J Nurs.

[8] Fathi Shykhi M, Shamsi M, Khorsandi M, Ranjbaran M (2015). The measurement of health belief model (HBM) Constructs in the Prevention of Accidents and Injuries in Children in Khorramabad, 2014. J Arak Univ Med Sci.

[9] Glanz K. Theory at a Glance: A Guide for Health Promotion Practice. 2nd ed. Bethesda: National Institutes of Health; 2005.

[10] Glanz K, Rimer BK, Viswanath K. Health Behavior and Health Education: Theory, Research, and Practice. 4th ed. San Francisco: Jossey-Bass; 2008.

[11] Nazareth M, Richards J, Javalkar K, Haberman C, Zhong Y, Rak E (2016). Relating health locus of control to health care use, adherence, and transition readiness among youths with chronic conditions, North Carolina, 2015. Prev Chronic Dis.

[12] Schilling S, Ritter VS, Skinner A, Yin HS, Sanders LM, Rothman RL (2020). Relationship between parental locus of control and childhood injury. J Prim Prev.

[13] Shojaeizadeh D, Hashemi SZ, Moeini B, Poorolajal J (2011). The effect of educational program on increasing cervical cancer screening behavior among women in Hamadan, Iran: applying health belief model. J Res Health Sci.

[14] Rahimi T, Faryabi R, Javadi A, Shojaei S (2017). Attitudes of women from Jiroft city about prevention of home injuries in children under 5 years using protection motivation theory in 2015. J Rafsanjan Univ Med Sci.

[15] Poorolajal J, Cheraghi P, Hazavehei SM, Rezapur-Shahkolai F (2012). Factors associated with mothers’ beliefs and practices concerning injury prevention in under five-year children, based on health belief model. J Res Health Sci.

[16] Vakili M, Momeni Z, Mohammadi M, Koohgardi M (2016). Epidemiological study of accidents in children under 6 years of Azadshahr Yazd in 2011. Pajouhan Sci J.

[17] Tsoumakas K, Dousis E, Mavridi F, Gremou A, Matziou V (2009). Parent’s adherence to children’s home-accident preventive measures. Int Nurs Rev.

[18] Akturk Ü, Erci B (2016). Determination of knowledge, attitudes and behaviors regarding factors causing home accidents and prevention in mothers with a child aged 0-5 years. J Educ Pract.

[19] Dehghani SS, Jafarzadeh S, Azadkhah F, Bazrafshan MR, Afzali Harsini P, Khani Jeihooni A (2019). Investigating the health belief model structures regarding the preventive factors from the occurrence of accidents and injuries in children under 5 years of age in Fasa city. Pol Ann Med.

[20] Lafta RK, Al-Shatari SA, Abass S (2013). Mothers’ knowledge of domestic accident prevention involving children in Baghdad city. Qatar Med J.

[21] Jaras M, Shamsi M, Moslemi A, Askari Moslehabadi A (2023). Factors related to the belief and performance of mothers in preventing injuries among children under 5 years of age: application of the health belief model. Payesh.

